# Effect of overexpression of *LPAAT* and *GPD1* on lipid synthesis and composition in green microalga *Chlamydomonas reinhardtii*

**DOI:** 10.1007/s10811-017-1349-2

**Published:** 2017-12-19

**Authors:** Chaogang Wang, Yi Li, Jun Lu, Xu Deng, Hui Li, Zhangli Hu

**Affiliations:** 1 Shenzhen Key Laboratory of Marine Bioresource and Eco-environmental Science, Guangdong Engineering Research Center for Marine Algal Biotechnology, College of Life Science, Shenzhen University, Shenzhen, 518060 People’s Republic of China; 2 School of Science and School of Interprofessional Health Studies, Faculty of Health and Environmental Sciences, Auckland University of Technology, Auckland, 1142 New Zealand; 3 Institute of Biomedical Technology, Auckland University of Technology, Auckland, 1142 New Zealand

**Keywords:** *Chlamydomonas reinhardtii*, Lipid content, Triacylglycerol synthesis, Transgenic algae, Intermittent heat shock

## Abstract

Biodiesel is an alternative energy source which has attracted increasing attention lately. Although algae-based biodiesel production has many benefits, it is still far from industrial application. Research suggests that improving lipid quality and production through genetic engineering of metabolic pathways will be the most promising way. To enhance lipid content, both lysophosphatidic acyltransferase gene (*c-lpaat*) and glycerol-3-phosphate dehydrogenase gene (*c-gpd1*), optimized according to the codon bias of *Chlamydomonas reinhardtii*, were inserted into the genomic DNA of model microalga *C. reinhardtii* by the glass bead method. Transgenic algae were screened by zeomycin resistance and RT-PCR. The transcription levels of inserted genes and the fatty acid content were significantly increased after intermittent heat shock. Most of all, the transcription levels of *c-lpaat* and *c-gpd1* were increased 5.3 and 8.6 times after triple heat shocks, resulting in an increase of 44.5 and 67.5% lipid content, respectively. Furthermore, the content of long-chain saturated fatty acids and monounsaturated fatty acids, especially C18 and C18:1t, notably increased, while unsaturated fatty acids dramatically decreased. The results of this study offer a new strategy combining genetic manipulation and intermittent heat shock to enhance lipid production, especially the production of long-chain saturated fatty acids, using *C. reinhardtii*.

**Electronic supplementary material:**

The online version of this article (10.1007/s10811-017-1349-2) contains supplementary material, which is available to authorized users.

## Introduction

Biodiesel is an alternative and relatively clean energy source which attracts increasing attention because the combustion of fossil fuels releases large amounts of CO_2_ and pollutants (Amaro et al. 2012; Mubarak et al. 2015). Microalgae are one of the most promising sources for the production of biodiesel as they possess short life cycle, perform photosynthesis, occupy less land, and absorb a large amount of CO_2_ (Mata et al. 2010; Talebi et al. 2013). The same as most plants, microalgae store lipid in the form of triacylglycerol (TAG), which is the main component of biodiesel (Mubarak et al. 2015). Found in microalgae, the lipid synthesis is mainly through two pathways, fatty acid synthesis and TAG synthesis, which happen in the chloroplast and the endoplasmic reticulum, respectively. Acetyl-coenzyme A (AcCoA) forms free fatty acids (FFAs) under the catalysis of Acetyl-CoA carboxylase (ACCase) and fatty acid synthase (FAS) (Liang and Jiang 2013; Kirchner et al. 2016). In addition, four enzymes are involved in the synthesis of TAG, including glycerol-3-phosphate dehydrogenase (GPDH), lysophosphatidic acyltransferase (LPAAT), diacylglycerol acyltransferase (DGAT), and glycerol-3-phosphate acyltransferase (GPAT) (Griffiths and Harrison 2009; Kirchner et al. 2016). Therefore, increasing the expression of those genes may achieve the aim of improving lipid content.

In the past few decades, researchers have screened algae through strain screening and mutagenesis with the aim of improving lipid content (Banerjee et al. 2016; Kirchner et al. 2016). Though research shows that lipid content of microalgae increases under nitrogen or phosphate starvation, the growth rate of algae is reduced (Hu et al. 2008; Griffiths and Harrison 2009; Himanshu et al. 2016). Nowadays, genetic engineering enables us to modify genes related to fatty acid synthesis to obtain strains with high lipid content in *Chlamydomonas*. However, expressing ACCase and FAS genes from plants in microalgae has not achieved the aim of significantly increasing oil content (Dunahay et al. 1995; Dehesh et al. 2001). Nevertheless, it is still hopeful to promote lipid content through enhancing key enzyme activity of TAG synthesis. In the meantime, transcriptome analysis of *Chlamydomonas reinhardtii* with high lipid accumulation reveals that *GPDH* and *LPAAT* are significantly upregulated, indicating a positive correlation between the transcription of those genes and cellular lipid accumulation (Lv et al. 2013; Fan et al. 2014). Introducing *CrGPDH* gene from *C. reinhardtii* into mutated yeast reveals that it causes higher glycerol production (Casais-Molina et al. 2016). When introducing glycerol-3-phosphate dehydrogenase gene (*GPD1*) from yeast to oil rapeseed, lipid content in the rapeseed is increased by 40% (Vigeolas et al. 2007). Moreover, overexpression of *LPAAT* in *Brassica napus* or *GPAT* in *Arabidopsis thaliana* enhances lipid content and TAG accumulation (Maisonneuve et al. 2010; Liang and Jiang 2013). The above results imply that changing fatty acid synthesis pathway or key enzyme transcription in chloroplast is not a plausible way to enhance lipid production in algae. On the other hand, enhancing key enzyme activity of TAG synthesis may promote TAG synthesis by accelerating the transport of FFAs from the chloroplast to the endoplasmic reticulum. Currently, there are a limited number of reports using genetic engineering to modify TAG synthesis pathway to enhance lipid production in microalgae. For example, it has been reported that overexpression of *DGAT* in *C. reinhardtii* has not significantly changed TAG composition or accumulation despite detecting a high level of transcription (La Russa et al. 2012). Therefore, more studies are needed to investigate the feasibility of enhancing lipid production in algae through genetic engineering of key enzymes in the TAG pathway.

Firstly, a strong and inducible promoter is needed to overexpress lipid-producing genes in *C. reinhardtii*. The Hsp70A-RBCS2 promoter has been previously studied and has shown that it could improve the transformation efficiency of a foreign gene and overexpress the foreign gene under 40 °C heat shock (Schroda et al. 2000, 2002). Hence, whether inserting this promoter in front of the lipid-producing gene combined with heat shock can improve gene expression and lipid production is worth investigating.

In this study, we used *C. reinhardtii* as a model to investigate the effect of *GPDH* and *LPAAT* on lipid production in microalgae. *Chlamydomonas reinhardtii* is a single-cell green alga which has been widely used as a biological model for photosynthesis and lipid metabolism (Ahmad et al. 2015). It has well-established genetic engineering systems in its nucleus, chloroplast, and mitochondria and has been used as a model to study exogenous gene expression and secondary metabolite synthesis. Here, *LPAAT* gene from *Brassica napus* and *GPD1* gene from *Saccharomyces cerevisiae* were resynthesized, according to the codon bias of *C. reinhardtii*, and then inserted into its genomic DNA to obtain gene overexpression. We also inserted Hsp70A-RBCS2 promoter and used intermittent heat shocks to find out whether lipid production in *C. reinhardtii* can be enhanced and to assess the feasibility of adjusting key enzymes of the TAG synthesis pathway through genetic engineering to enhance lipid production.

## Materials and methods

### Strains and growth conditions

*Chlamydomonas reinhardtii* strain CC-849 was purchased from the Chlamydomonas Center in the USA and maintained in Tris-acetate-phosphate (TAP) medium (Gorman and Levine 1965), under the temperature of 22 °C and continuous light at 20 μmol photons m^−2^ s^−1^ (normal condition). *Escherichia coli* TOP10 was taken from the bacterial stocks in our laboratory. Plasmid pH124 carrying the *ble* conferring zeomycin and phleomycin resistance in host cells was constructed and kept in our laboratory (Wu et al. 2008). Tran^clpaat^ and Tran^cgpd1^ were transgenic algae with introduced *c-lpaat* and *c-gpd1*, respectively.

To apply heat shock (HS), 400 mL cells (wild-type (WT) and transgenic algae) in the late logarithmic phase were incubated at 40 °C and light intensity of 20 μmol photons m^−2^ s^−1^ for 15 min. Then, the conditions were restored to normal. As for multiple HSs, a 4-h recovery period was placed between each HS. The sampling time points for a single heat shock (HS1) were as follows: before HS, 0 min after HS, and 30, 60, 90, and 120 min after recovery. The sampling time points for triple heat shocks (HS3) were as follows: before HS, 0 min after each HS, and 30, 120, and 240 min after each recovery. The WT strain CC-849 without *c-lpaat* and *c-gpd1* was used as the control. After the treatment, algal cells (WT and transgenic algae) were subjected to DNA/RNA extraction and GC-MS analysis.

### Construction of vector

**Fig. 1:**
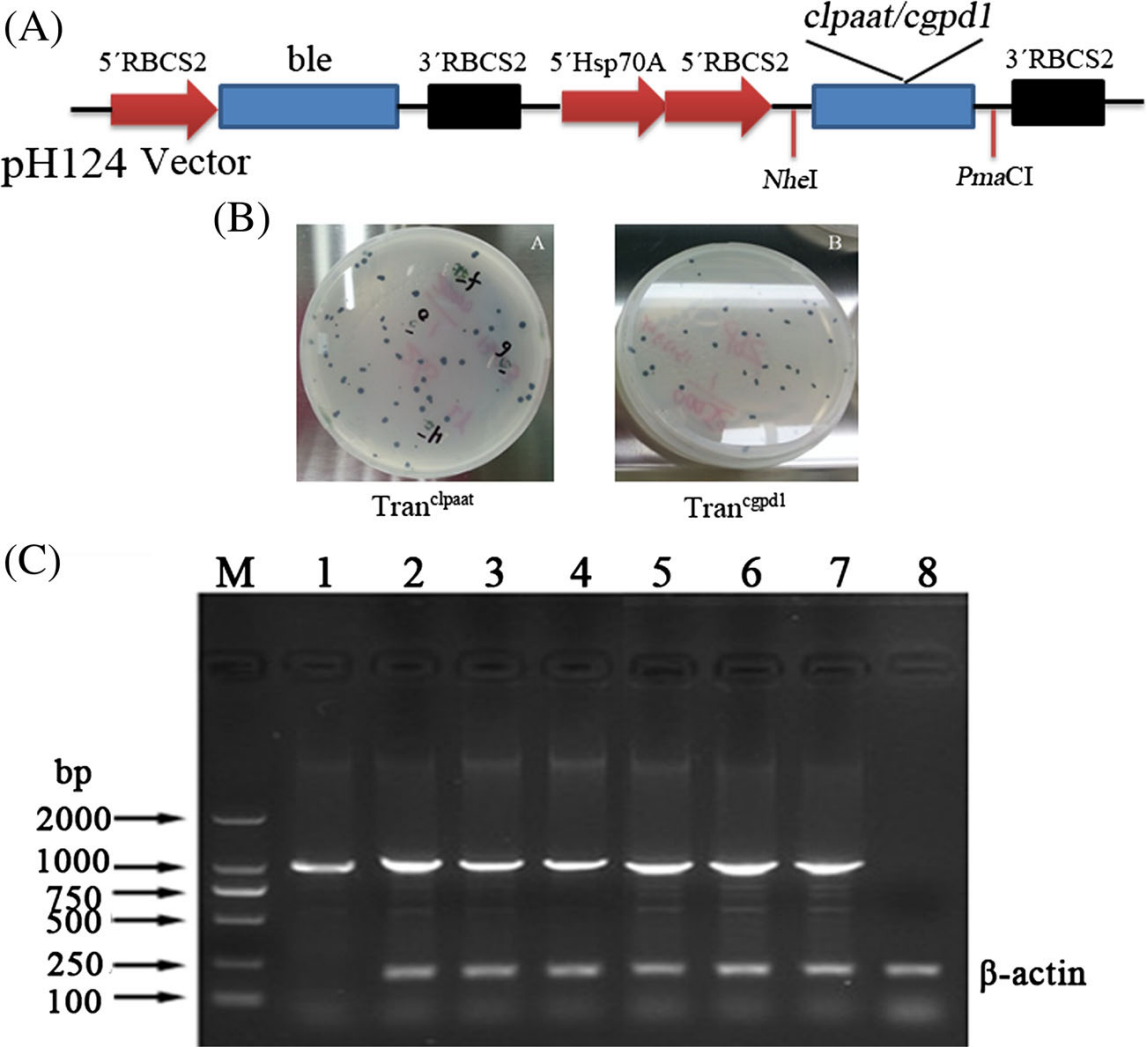
Obtainment of transgenic algae with *c-lpaat* and *c-gpd1*. **a** Construction of transforming plasmids. *c-lpaat* and *c-gpd1* were inserted into pH124 that contained the ampicillin- and zeomycin-resistant genes and a strong heat-inducible promoter (*Hsp70A* promoter). **b** Screening transformants through zeomycin resistance (10 μg mL^−1^). Green clones were visible after 2–3 weeks, and transformants were kept in TAP containing 10 μg mL^−1^ zeomycin. **c** RT-PCR verification of transgenic algae with *c-lpaat* and *c-gpd1*. Specific fragments of 1176 bp were amplified from the total RNA of transformants. M DL 2000, 1 positive control, PCR fragment of 1176 bp, 2–4 transgenic algae with *c-lpaat*, 5–7 transgenic algae with *c-gpd1*, 8 negative control, PCR fragment of *β-actin* from wild-type CC-849. No *c-lpaat* and *c-gpd1* products were detected in WT

*LPAAT* gene from *Brassica napus* (GenBank: AY616009) and *GPD1* gene from *Saccharomyces cerevisiae* (GenBank: Z24454) were resynthesized as *c-lpaat* and *c-gpd1* according to the codon bias of *C. reinhardtii* and cloned in plasmid pUC57 (Sangon Biotech Co., Ltd., China). Fragments of *c-lpaat* and *c-gpd1* were obtained after cleavage, using restriction endonucleases *Nhe* I and *Pma*C I, and then were inserted into pH124 vector to obtain pH124-*c-lpaat* and pH 124-*c-gpd1*, respectively. The pH124 vector already contained *Hsp70A-RBCS2* promoter, *RBCS2* terminator, and *ble* gene (Fig. 1a).

### Nuclear transformation of *C. reinhardtii*

Genetic transformation of *C. reinhardtii* CC-849 was carried out using the “glass bead method,” according to the protocol described by Kindle (1990) and Wang et al. (2017).

### RNA extraction

Total RNAs were isolated using a RNA extraction kit (FAST200) (Fastagen Biotechnology Co., Shanghai, China) with DNase I treatment to eliminate possible DNA contamination according to the manufacturer’s instructions.

### RT-PCR verification of transgenic algae

Total RNAs extracted from WT *C. reinhardtii* and transgenic lines were used as the templates for RT-PCR reactions. The primer sets *c-lpaat*-F/*c-lpaat*-R and *c-gpd1*-F/*c-gpd1*-R were used to amplify the *c-lpaat* and *c-gpd1* genes. The *β-actin* gene (as the internal control) was amplified with the primer sets Actin-F/Actin-R (Table 1). PCR program was set as follows: initial denaturation at 95 °C for 2 min, followed by 30 cycles of incubation at 95 °C for 30 s, at 60 °C for 15 s, at 72 °C for 15 s, and a final extension at 72 °C for 5 min.

**Table 1:** List of primer sets used in RT-PCR

Primer name	Primer sequences	Product (bp)
*c-lpaat*-F	5′ CCAAGGTGGCTCGTGACTC 3′	1176
*c-lpaat*-R	5′ ACTCGCCTCTGTGCCTGTT 3′	
*c-gpd1*-F	5′ CGCCGACCGCCTGAACCT 3′	1176
*c-gpd1*-R	5′ CGCCGACCGCCTGAACCT 3′	
Actin-F	5′ ACCCGTGCTGACTG 3′	240
Actin-R	5′ ACGTTGAAGGTCTCGAACA 3′	

### Gene expression profiling: real-time RT-PCR

Total RNAs in WT and transgenic algae were extracted from cells in the late logarithmic phase. Real-time RT-PCR analysis was performed on an ABI PRISM 7900 sequence detection system (Applied Biosystems, USA) following the protocol previously described using *β-actin* gene as the internal control and SYBR Premix Ex Taq kit (Takara, Japan). To obtain cDNA, 1-μg extracted RNA was reversely transcribed into cDNA and then, an equal amount of cDNA was selected as template to perform qRT-PCR. Primer sets for *c-lpaat*, *c-gpd1*, and *β-actin* genes are described in Table 2. PCR conditions were as follows: one step of 95 °C incubation for 30, followed by 40 cycles of 95 °C for 5 and 55 °C for 30s. Data with an *R* ^2^ above 0.998 was analyzed using the 2^−ΔΔCt^ program (Lei et al. 2012). Three technical replicates and two biological replicates were used. The transcription value of *c-lpaat* and *c-gpd*1 before HS was defined as 1, and then, we obtained the other transcription values and expressed them as relative ratios compared against the before-HS value.

**Table 2:** List of primer sets used in real-time RT-PCR

Gene name	Primer name	Primer sequences
*c-lpaat*	Q-*c-lpaat*-F	5′ CCTGTGGCTGGAGCTGGTGT 3′
	Q-*c-lpaat*-R	5′ ATGTCCGAGCGGTGGTTGG 3′
*c-gpd1*	Q-*c-gpd1*-F	5′ GTGGTGGCCGAGAACTGCA 3′
	Q-*c-gpd1*-R	5′ TGGTGGCGGGTGTTGATG 3′
*β-actin*	Actin-F	5′ ACCCGTGCTGCTGATG 3′
	Actin-R	5′ACGTTGAAGGTCTCGAACA 3′

### Fatty acid methyl ester transformation and FAME analysis

Total lipid extraction was performed as previously described (Lu et al. 2012) with slight modifications. Freeze-dried algal powder of 15 mg was weighed. C_19_ (500 μg mL^−1^, ANPEL Laboratory Technologies (Shanghai) Inc.) was added into the sample as the internal standard. Qualification and quantification of fatty acid methyl esters (FAMEs) were performed on a Thermo Trace GC Ultra gas chromatograph coupled to Thermo Polaris Q mass spectrometry which was equipped with a HP-5MS column (30 m × 0.32 mm id, film thickness 0.25 μm). The temperature of the injector was maintained at 250 °C. Helium was used as the carrier gas, ions were generated by a 70-V electron beam, and the mass range scanned was 50 to 650 *m*/*z* at a rate of 2 scans s^−1^. The oven temperature for FAME analysis was initially maintained at 70 °C for 4 min, followed by a temperature increment rate of 5 °C min^−1^ to 195 °C; held for 5 min, followed by 3 °C min^−1^ increase to 205 °C; held for 2 min, followed by 8 °C min^−1^ increment to 230 °C; and then held for 1 min. GC-MS transmitting line temperature was maintained at 250 °C. Peak identification was performed by matching the mass spectra of each compound with the National Institute of Standards and Technology mass spectral library. Automatic peak deconvolution was processed with Masslynx software (V4.1, Waters Corp.,USA) (Lei et al. 2012). The datasets of FAME profiling for further analysis were obtained by normalization against the internal standard in the same chromatogram.

### Protein extraction and quantification

When algal growth reached the exponential phase, algal culture was collected and centrifuged at 5000×*g* and 22 °C for 5 min. The supernatant was discarded, and plant total protein extraction kit (Sangon Biotech (Shanghai) Co., Ltd.) was used to extract total protein from the remaining algal cells. Bovine serum albumin (BSA) standard curve was constructed to measure the protein content according to the above protein-measuring kit’s instruction.

### Statistical analysis

All experiments were repeated three times independently, and data were recorded as the mean with standard deviation (SD). For gene expression experiments, quantitative real-time PCR analysis was performed using the BioRAD iQ5 software. For each gene, the fold change was expressed as the mean ± SD (% control) and was calculated using the standard curve with approximation corrected for primer efficiency and normalized to housekeeping gene *β-actin* expression values. Statistical analyses were performed using the Student’s *t* test (SPSS19.0). For all analyses, a *p* value < 0.05 was considered statistically significant.

## Results

### Vector construction and transgenic alga obtainment

The *LPAAT* and *GPD1* genes were resynthesized according to the codon bias of *C. reinhardtii* to obtain *c-lpaat* and *c-gpd1* genes suitable for *C. reinhardtii* expression (Stevens et al. 1996) (see supplementary material 1). When treating with 42 °C HS, both *c-lpaat* and *c-gpd1* genes under the control of *Hsp70A* promoter were overexpressed (Fig. 1a). Through zeomycin resistance and RT-PCR screening, transgenic algal cells were obtained. Transgenic algae inserted with *c-lpaat* and *c-gpd1* were named as Tran^clpaat^ and Tran^cgpd1^, respectively (Fig. 1b, c). Transformation efficiency by the glass bead method was 8.4 × 10^−7^ and 4 × 10^−7^ cells mL^−1^ for *c-lpaat* and *c-gpd1*, respectively.

### Overexpressing *c-lpaat* and *c-gpd1* in transgenic *C. reinhardtii*

**Fig. 2:**
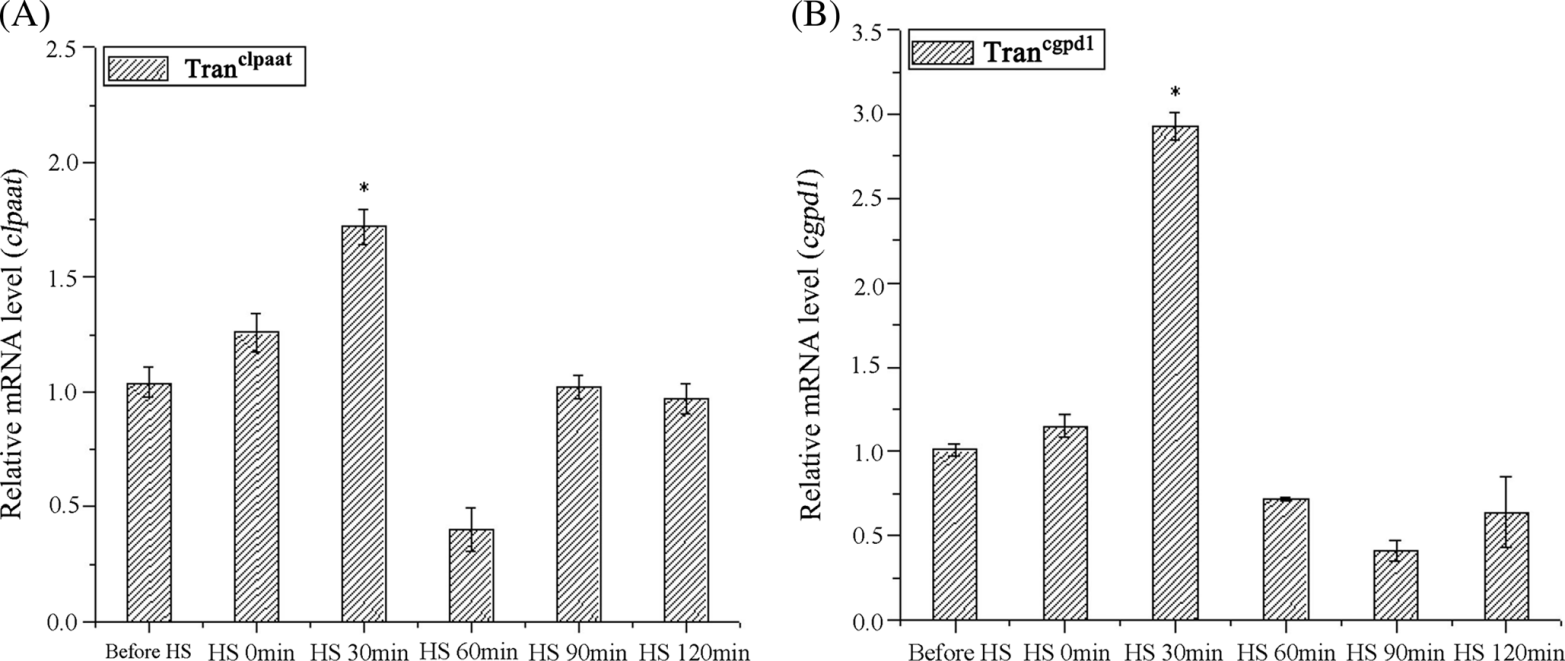
Single heat shock-enhanced transcription level of transformed genes in transgenic *C. reinhardtii* (HS heat shock, under 40 °C and 20 μmol photons m^−2^ s^−1^ light for 15 min, HS 0 min sampling immediately after heat stimulation. *P < 0.05 compared with before heat shock)

Both *c-lpaat* and *c-gpd1* controlled by *Hsp70A* promoter were overexpressed under HS. The transcription levels of target genes were unchanged during HS but increased quickly after algae cells were cooled down to the normal condition and reached the highest level after 30 min, with *c-lpaat* increased 1.93 times and *c-gpd1* increased 2.98 times (Fig. 2a, b).

**Fig. 3:**
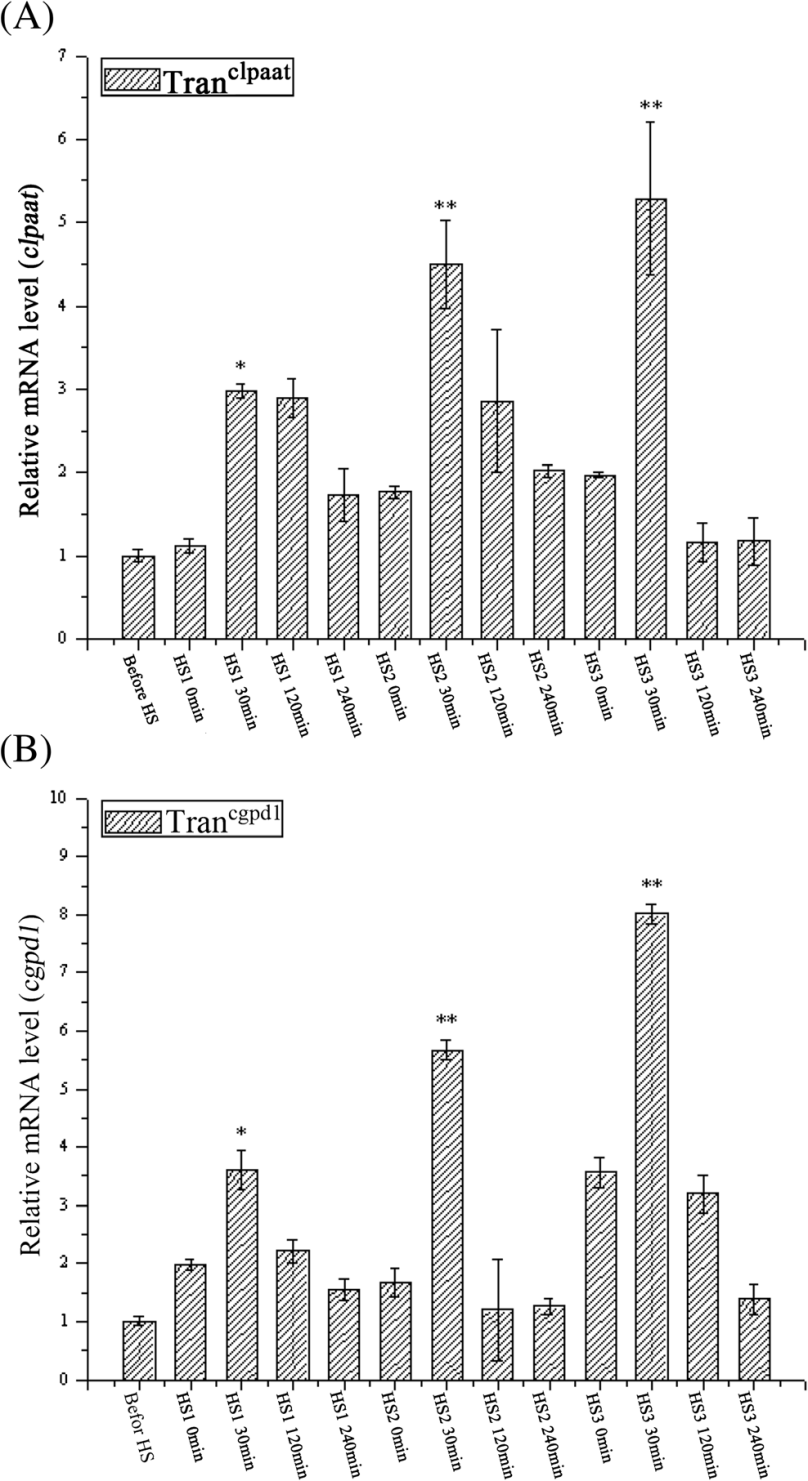
Triple heat shocks enhanced **a** *c-lpaat* and **b** *c-gpd1* gene transcriptions in transgenic *C. reinhardtii*. (*P < 0.05; **P < 0.01 compared with before heat shock)

Interestingly, the transcription levels of *c-lpaat* and *c-gpd1* were further improved when treated with intermittent HS. For instance, the transcription levels of *c-lpaat* in Tran^clpaat^ were increased 3.01, 4.46, and 5.3 times after three HSs, respectively (Fig. 3a). The transcription levels of *c-gpd1* in Tran^cgpd1^ were increased 3.6, 5.42, and 8.58 times, respectively (Fig. 3b). The transcription levels of both genes reached the peak at 30 min after HS (Fig. 3a, b). Based on the data analysis, it was confirmed that the transcription levels of target genes were dramatically increased after HS.

### Heat shock enhances fatty acid content in transgenic *C. reinhardtii*

**Fig. 4:**
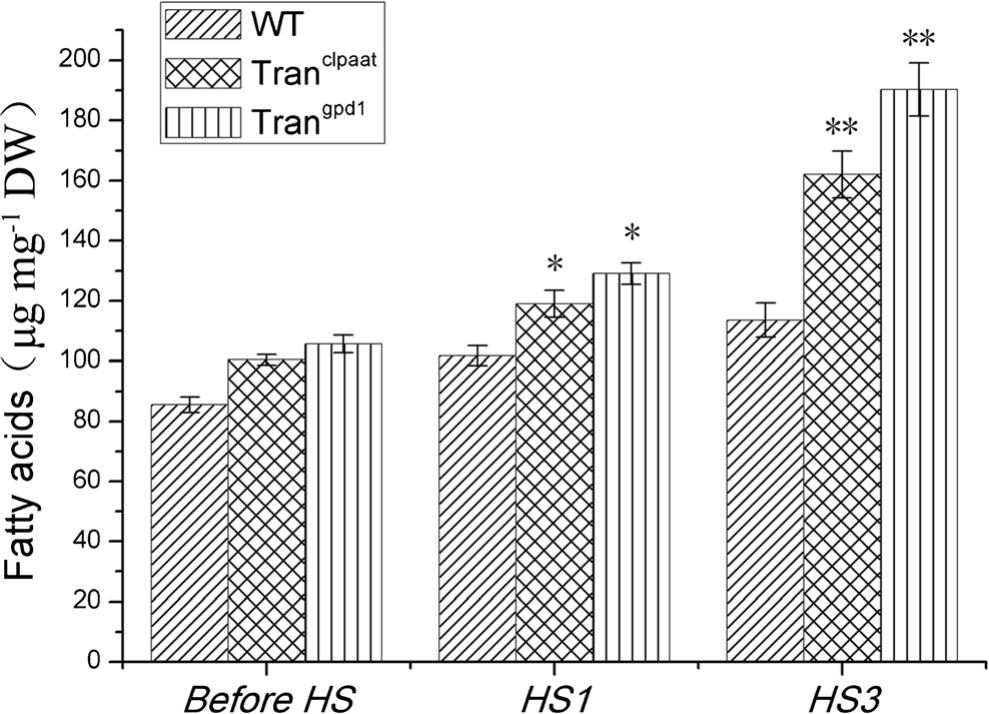
Lipid content change of transgenic *C. reinhardtii* after heat shock (HS1 and HS3 stand for single heat shock and triple heat shocks, respectively.). After heat shock, total fatty acids of Tran^clpaat^ and Tran^cgpd1^ significantly increased when compared with wild-type CC-849

As shown above, transcription levels of *c-lpaat* and *c-gpd1* in transgenic *C. reinhardtii* could be enhanced significantly after HS. Fatty acids (FAs) were extracted from WT and all transgenic algal cells under normal condition and after HS. Compared to the WT, under the normal culture condition, the total fatty acid (TFA) contents of transgenic alga strains including Tran^clpaat^ and Tran^cgpd1^ strains increased by 17.4 and 23.6%, respectively. The HS treatment further increased the TFA in transgenic algae. The TFA contents in WT were 101.79 and 113.57 μg mg^−1^ dry weight (DW) after one heat shock (HS × 1) and three heat shocks (HS × 3), respectively. TFA of Tran^clpaat^ and Tran^cgpd1^ increased 16.8 and 26.7%, respectively, after HS × 1, compared with WT cells treated with HS × 1 (Fig. 4). Transgenic algae had significantly higher TFA than WT cells after HS × 3, with an increase of 44.5% (Tran^clpaat^) or 67.5% (Tran^cgpd1^) (Fig. 4). These results were consistent with the high level of gene transcriptions of *c-lpaat* and *c-gpd1*, indicating that overexpression of those genes could enhance lipid accumulation in transformed *C. reinhardtii* cells.

### Change in lipid composition of transgenic algae

The fatty acid profile in algae was analyzed by using GC-MS. WT and transformed algae have similar fatty acids as shown by GC-MS peaks (data not shown). The increase in TFA content of transgenic algae was contributed by the increase of nearly all types of fatty acids. For example, C18:1t in Tran^clpaat^ and Tran^cgpd1^ cells had increases ranging from 177.3 to 270.9% after HS × 1, compared to the WT. The most abundant component is still C18:3n3. In Tran^clpaat^, C16:0, C18:0, and C18:2t had increases ranging from 33 to 38%. As to Tran^cgpd1^, C16:0, C16:1, C18:0, and C18:2t had increases ranging from 43 to 73% (see Table 3 for more details).

**Table 3:** Change of algal fatty acid content and composition after one heat shock

Fatty acid	Wild type (μg mg^−1^ DW)	Tran^clpaat^ (μg mg^−1^ DW)	Increase (%)	Tran^cgpd1^ (μg mg^−1^ DW)	Increase (%)
16:0	15.05 ± 0.06	20.03 ± 0.09*	33.09	21.87 ± 0.08*	45.32
16:1	1.98 ± 0.16	1.99 ± 0.51	0.51	2.85 ± 0.19*	43.94
16:4	27.71 ± 0.38	29.03 ± 0.11	4.76	31.25 ± 0.40	12.78
18:0	1.55 ± 0.06	2.15 ± 0.06*	38.71	2.27 ± 0.07*	46.45
18:1t	1.10 ± 0.12	3.05 ± 0.09**	177.27	4.08 ± 0.13**	270.9
18:2t	7.47 ± 0.35	10.06 ± 0.13*	34.67	12.96 ± 0.27*	73.49
18:3	9.83 ± 0.09	10.68 ± 0.29	8.65	10.78 ± 0.16	9.66
18:3n3	37.10 ± 0.08	41.99 ± 0.18	13.18	43.01 ± 0.29	15.93

Average of all the observations of four repeated tests, in the form of mean ± standard error of representation; wild-type CC-849 represents a control. All data unit is in milligram per gram (dry weight). Statistical analysis software SPSS 19.0

After HS × 3, in Tran^clpaat^ cells, the content of C18:0 and C18:1t fatty acids increased by 355.3 and 220.1%, respectively, compared to the WT. In Tran^cgpd1^ cells, the content of C18:0 and C18:1 t fatty acids increased by 428.2 and 394.2%, respectively, compared to the WT (see Table 4 for more details). Results showed that the increased fatty acids were mainly C18 and the most abundant component is still C18:3n3. However, in Tran^clpaat^ and Tran^cgpd1^ cells, C18:3n3 percentage decreased from 34 to 27%, while C18:0 increased from 2 to 7%. The content of C16:0 and C16:1 fatty acids increased after HS × 3 in Tran^clpaat^ and Tran^cgpd1^. Hence, it is clear that introducing *c-lpaat* and *c-gpd1* to *C. reinhardtii* can significantly increase cellular lipid accumulation, in particular enhancing the production of monounsaturated fatty acids and long-chain saturated fatty acids, which could be beneficial for biodiesel production.

**Table 4:** Change of algal fatty acid content and composition after triple heat shocks

Fatty acid	Wild type (μg mg^−1^ DW)	Tran^clpaat^ (μg mg^−1^ DW)	Increase (%)	Tran^cgpd1^ (μg mg^−1^ DW)	Increase (%)
16:0	18.05 ± 0.10	29.83 ± 0.06*	65.26	35.39 ± 0.04*	96.07
16:1	2.98 ± 0.06	4.55 ± 0.51*	52.68	6.93 ± 0.21*	132.55
16:4	29.71 ± 0.78	40.99 ± 0.11	37.97	39.65 ± 1.16	33.46
18:0	2.55 ± 0.22	11.61 ± 0.36**	355.29	13.47 ± 0.25**	428.24
18:1t	1.89 ± 0.06	6.05 ± 0.09**	220.11	9.34 ± 0.46**	394.18
18:2t	8.47 ± 0.13	13.06 ± 0.13*	54.19	18.72 ± 0.37*	121.02
18:3	10.83 ± 0.16	11.98 ± 0.50	10.62	15.36 ± 0.11*	41.83
18:3n3	39.09 ± 0.19	44.04 ± 0.07	12.66	51.37 ± 0.21	31.41

Average of all the observations of four repeated tests, in the form of mean ± standard error of representation; wild-type CC-849 represents a control. All data unit is in milligram per gram (dry weight). Statistical analysis software SPSS 19.0

### Introducing *c-lpaat* and *c-gpd1* to *C. reinhardtii* reduces protein synthesis

The growth of WT and transgenic algae was similar, and the highest concentration was around 5.8 × 10^6^ cells mL^−1^ for all strains, indicating that the transformed genes had no effect on the growth of algal cells (see supplementary material 2).

**Fig. 5:**
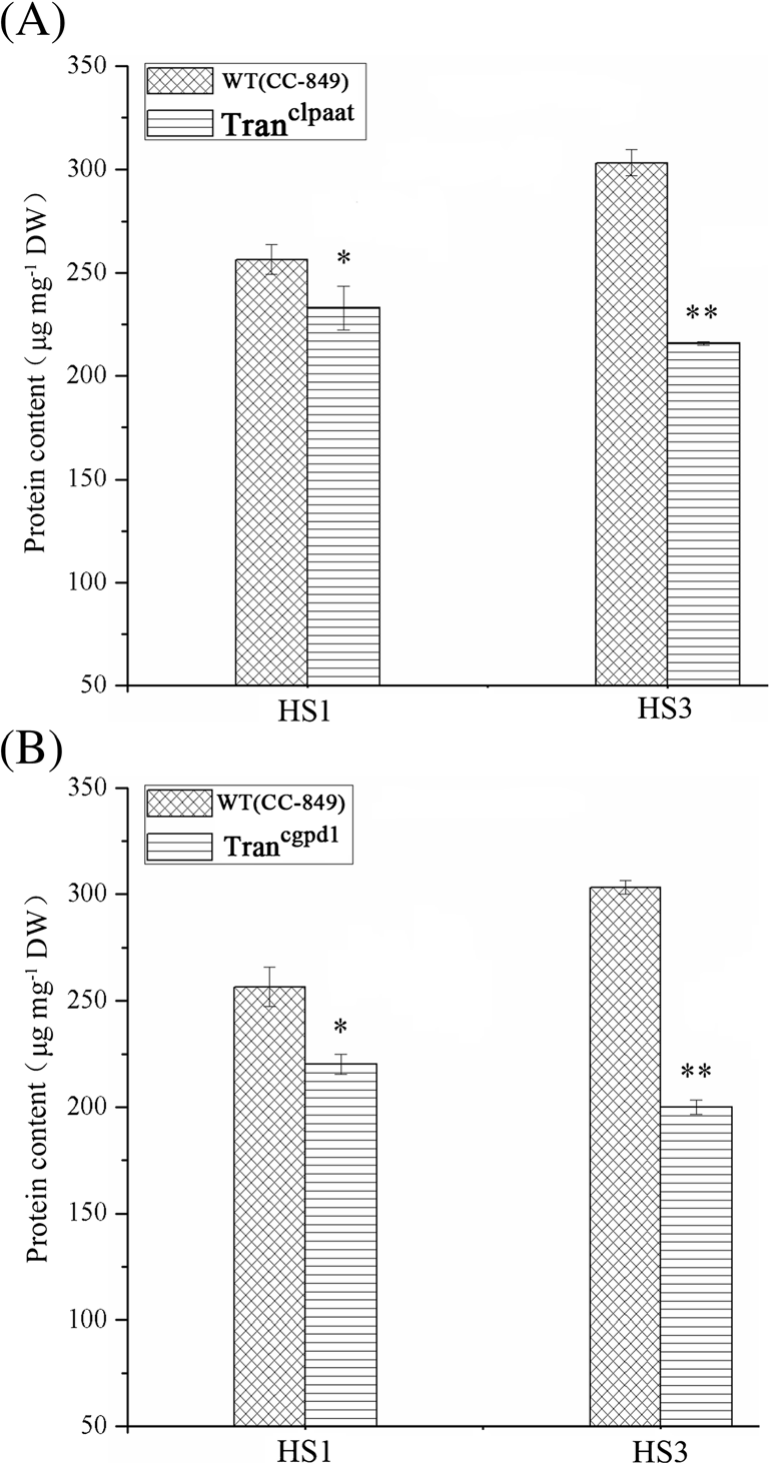
Protein content change after heat shock in **a** Tran^clpaat^ and **b** Tran^cgpd1^ cells. (HS heat shock, under 40 °C and 20 μmol photons m^−2^ s^−1^ light for 15 min, HS 0 min sampling immediately after heat stimulation. *P < 0.05 compared with the wild type)

*Chlamydomonas reinhardtii* produces protein and lipid needed for cell function via photosynthesis. Therefore, the carbon amount is relatively fixed and kept in balance in the cellular system. There is a competitive relationship between protein and lipid syntheses in alga cells, and there are reports showing that inhibition of phosphoenolpyruvate carboxylase can enhance cellular fatty acid content in algae (Deng et al. 2014). After HS × 1, protein content of Tran^clpaat^ and Tran^cgpd1^cells decreased by 9.2 and 14.1%, respectively, compared to WT. Furthermore, protein contents of Tran^clpaat^ and Tran^cgpd1^ cells decreased by 29 and 34%, respectively, after HS × 3 (Fig. 5a, b). Interestingly, the protein content in WT showed increase after HS × 1 and HS × 3, suggesting that HS in this study had no negative effect on the growth of algae.

All of the above demonstrate that introducing *c-lpaat* and *c-gpd1* to *C. reinhardtii* changed carbon flow direction, which increased lipid production but reduced protein synthesis. Introducing *c-gpd1* rendered higher efficiency than introducing *c-lpaat*, as Tran^cgpd1^ had 23% more lipid content but only had 5.2% lower protein level.

## Discussion

Recent reports show that lipid production in *C. reinhardtii* can be enhanced under nitrogen starvation (James et al. 2011). Transcriptome analysis shows that the expression of more than 2500 genes is upregulated, among which the transcription of enzyme genes in TAG pathway is significantly increased. One of them, *GPDH*, increases 5.8 times, and another gene, *LPAAT*, increases 3.6 times (Lv et al. 2013). GPDH catalyzes the synthesis of glycerol-3-phosphate, and LPAAT catalyzes the synthesis of phosphatidic acid, both supply substrates for TAG synthesis (Deng et al. 2011). Hence, in this study, those two genes were introduced into *C. reinhardtii* to investigate their ability to enhance lipid production. High-level transcription of *LPAAT* and *GPD1* was detected, with increases of 5.3 and 8.6 times. We successfully used intermittent HS strategy to enhance lipid production for up to 67.5% more than the WT.

Currently known key TAG synthesis enzymes are GPDH, GPAT, LPAAT, and DGAT (Lv et al. 2013). Inhibition of *LPAAT* and *DGAT* at transcription levels results in lipid reduction (Zhang et al. 2005; Lv et al. 2013). On the other hand, introducing and overexpressing genes related to TAG synthesis cause lipid content to increase (Jain et al. 2000; Zhang et al. 2005). Those results suggest that key enzymes of TAG synthesis play important roles in lipid production. Our approach of introducing *LPAAT* and *GPD1* into *C. reinhardtii* is capable of enhancing lipid production significantly under HS. This approach has the potential to be used in high-oil-production algae, such as diatoms. Successful application of our strategy may reduce the cost of biodiesel production.

In our study, *Hps70A* promoter has been used to drive *c-gpd1* and *c-lpaat* expressions. This promoter can greatly enhance gene expression under heat stimulation (Schroda et al. 2002). Previous reports show that the transcription level of target genes increases 3–26 times when employing *Hsp70A* promoter to express them (Schroda et al. 2000, 2002; Wu et al. 2008). We attempted intermittent multiple HS strategy to increase gene expression without affecting the growth of algal cells. Our intermittent triple-HS method has been proven to be successful, where gene expression has been increased significantly compared with a single HS. Since mRNA is unlikely to accumulate after expression and HS and TATA-binding factors can bind to the *cis*-elements of *Hsp70A* promoter such as HSE sequence and TATA-box (Schroda et al. 2000, 2002), we suggest that after the first HS, those regulatory proteins should be retained in the cells, which bind to the promoter faster at the second and third HSs, which in turn results in fast enhancement of gene expression. The enhanced expression of *c-gpd1* and *c-lpaat* increases the TAG synthesis. The 67.5% increase in lipid production resulting from the above is significantly higher than previous reports in other microalgae (La Russa et al. 2012; Ibáñez-Salazar et al. 2014; Kang et al. 2015).

In *C. reinhardtii*, the fatty acids are fairly evenly split between 16-carbon chains (two thirds of which are C16:0) and 18-carbon chains (predominantly C18:3). Non-saturated fatty acid content is higher than saturated fatty acid content, and the main fatty acids include C16:0, C16:4, and C18:3n3 (James et al. 2011). Change of fatty acid synthesis enzymes may change the composition of fatty acids as well. In particular, GPDH catalyzes dihydroxyacetone phosphatein to glycerol-3-phosphate, while LPAAT catalyzes the synthesis of phosphatidic acid (La Russa et al. 2012). When introducing diacylglycerol acyltransferase gene from *B. napus* to *C. reinhardtii*, there is no major change in the main fatty acid components. However, the levels of saturated fatty acids in the transformed algae decrease to about 7% while unsaturated fatty acids increase proportionately. Polyunsaturated fatty acids, especially α-linolenic acid, increase up to 12% in the transformed line. Interestingly, C18:1–3 slightly decreases in the *DAGAT* mutant strain in *C. reinhardtii*, while C16:0 slightly increases (La Russa et al. 2012). In yeast with introduced *LPAAT*, the proportion of C12:0 and C14:0 fatty acids increases, while in *N. tabacum* with introduced *LPAAT*, that proportion actually decreases (Yuan et al. 2015). Hence, the same gene expressed in different species may result in a different outcome. There is little information about the role of TAG synthesis enzymes in microalga lipid production. In this study, we have shown that with introduced *LPAAT* or *GPD1*, saturated and monounsaturated fatty acids (C16:0, C18:0, C18:1t, and C18:2 t) have increased significantly. This is consistent with a previous report (Mentewab and Stewart 2005). We also have observed that polyunsaturated fatty acids decreased significantly. Therefore, our genetic manipulation has increased long-chain saturated fatty acids, predominantly C16–C18, which make genetically modified *C. reinhardtii* an attractive source for biodiesel production with relatively low costs.

In conclusion, we have established a *C. reinhardtii* genetically modified model that uses *Hsp70A* promoter to overexpress *LPAAT* gene from *B. napus* and *GPD1* gene from *S. cerevisiae*. Applying intermittent multiple-HS strategy, this model can increase lipid production by up to 67.5% and the introduced *c-lpaat* and *c-gpd1* gene expressions can be enhanced by 5.3 and 8.6 times, respectively. We also have proven that overexpression of *c-lpaat* and *c-gpd1* genes is the cause of high lipid production, which in the meantime reduces protein content. Our results suggest that it is possible to create new strains of microalgae with high lipid production ability, and this method can reduce the cost of biodiesel production, which has high technological and economic potentials.

## Electronic supplementary material

ESM 1 (DOCX 15.5 kb)

ESM 2 (DOCX 94.3 kb)

ESM 3 (DOCX 15.9 kb)
