# Supplementary material for: Effect of overexpression of LPAAT and GPD1 on lipid synthesis and composition in green microalga Chlamydomonas reinhardtii
Source: J Appl Phycol. 2017 Dec 19;30(3):1711–9. doi: 10.1007/s10811-017-1349-2 (PMC5982436; doi:10.1007/s10811-017-1349-2)
Supplement: Supplementary file 1 — (DOCX 15.5 kb) [file 10811_2017_1349_MOESM1_ESM.docx]

**Supplementary Material 1**

Optimized sequence of *c-lpaat*

1 ATGGC CATGG CCGCC GCCGC CGTGA TCGTG CCCCT GGGCA TCCTG

46 TTCTT CATCT CGGGC CTGGT GGTGA ACCTG CTGCA GGCCG TGTGC

91 TACGT GCTGA TCCGC CCCCT GTCGA AGAAC ACCTA CCGCA AGATC

136 AACCG CGTGG TGGCC GAGAC CCTGT GGCTG GAGCT GGTGT GGATC

181 GTGGA CTGGT GGGCC GGCGT GAAGA TCCAG GTGTT CGCCG ACGAC

226 GAGAC CTTCA ACCGC ATGGG CAAGG AGCAC GCCCT GGTGG TGTGC

271 AACCA CCGCT CGGAC ATCGA CTGGC TGGTG GGCTG GATCC TGGCC

316 CAGCG CTCGG GCTGC CTGGG CTCGG CCCTG GCCGT GATGA AGAAG

361 TCGTC GAAGT TCCTG CCCGT GATCG GCTGG TCGAT GTGGT TCTCG

406 GAGTA CCTGT TCCTG GAGCG CAACT GGGCC AAGGA CGAGT CGACC

451 CTGAA GTCGG GCCTG CAGCG CCTGA ACGAC TTCCC CCGCC CCTTC

496 TGGCT GGCCC TGTTC GTGGA GGGCA CCCGC TTCAC CGAGG CCAAG

541 CTGAA GGCCG CCCAG GAGTA CGCCG CCACC TCGCA GCTGC CCGTG

586 CCCCG CAACG TGCTG ATCCC CCGCA CCAAG GGCTT CGTGT CGGCC

631 GTGTC GAACA TGCGC TCGTT CGTGC CCGCC ATCTA CGACA TGACC

676 GTGGC CATCC CCAAG ACCTC GCCCC CCCCC ACCAT GCTGC GCCTG

721 TTCAA GGGCC AGCCC TCGGT GGTGC ACGTC CACAT CAAGT GCCAC

766 TCGAT GAAGG ACCTG CCCGA GTCGG AGGAC GAGAT CGCCC AGTGG

811 TGCCG CGACC AGTTC GTGGC CAAGG ACGCC CTGCT GGACA AGCAC

856 ATCGC CGCCG ACACC TTCCC CGGCC AGAAG GAGCA GAACA TCGAC

901 CGCCC CATCA AGTCG CTGGC CGTGG TGGTG TCGTG GGCCT GCCTG

946 CTGAC CCTGG GCGCC ATGAA GTTCC TGCAC TGGTC GAACC TGTTC

991 TCGTC GCTGA AGGGC ATCGC CCTGT CGGCC CTGGG CCTGG GCATC

1036 ATCAC CCTGT GCATG CAGAT CCTGA TCCGC TCGTC GCAGT CGGAG

1081 CGCTC GACCC CCGCC AAGGT GGCCC CCGCC AAGCC CAAGG ACAAG

1126 CACCA GTCGG GCTCG TCGTC GCAGA CCGAG GTGGA GGAGA AGCAG

1171 AAGTA A-CACGTG (*Pma*C I)-AAACGCG

Optimized sequence of *c-gpd1*

1 ATGTC GGCCG CCGCC GACCG CCTGA ACCTG ACCTC GGGCC ACCTG

46 AACGC CGGCC GCAAG CGCTC GTCGT CGTCG GTGTC GCTGA AGGCC

91 GCCGA GAAGC CCTTC AAGGT GACCG TGATC GGCTC GGGCA ACTGG

136 GGCAC CACCA TCGCC AAGGT GGTGG CCGAG AACTG CAAGG GCTAC

181 CCCGA GGTGT TCGCC CCCAT CGTGC AGATG TGGGT GTTCG AGGAG

226 GAGAT CAACG GCGAG AAGCT GACCG AGATC ATCAA CACCC GCCAC

271 CAGAA CGTGA AGTAC CTGCC CGGCA TCACC CTGCC CGACA ACCTG

316 GTGGC CAACC CCGAC CTGAT CGACT CGGTG AAGGA CGTGG ACATC

361 ATCGT GTTCA ACATC CCCCA CCAGT TCCTG CCCCG CATCT GCTCG

406 CAGCT GAAGG GCCAC GTCGA CTCGC ACGTC CGCGC CATCT CGTGC

451 CTGAA GGGCT TCGAG GTGGG CGCCA AGGGC GTGCA GCTGC TGTCG

496 TCGTA CATCA CCGAG GAGCT GGGCA TCCAG TGCGG CGCCC TGTCG

541 GGCGC CAACA TCGCC ACCGA GGTGG CCCAG GAGCA CTGGT CGGAG

586 ACCAC CGTGG CCTAC CACAT CCCCA AGGAC TTCCG CGGCG AGGGC

631 AAGGA CGTGG ACCAC AAGGT GCTGA AGGCC CTGTT CCACC GCCCC

676 TACTT CCACG TCTCG GTGAT CGAGG ACGTG GCCGG CATCT CGATC

721 TGCGG CGCCC TGAAG AACGT GGTGG CCCTG GGCTG CGGCT TCGTG

766 GAGGG CCTGG GCTGG GGCAA CAACG CCTCG GCCGC CATCC AGCGC

811 GTGGG CCTGG GCGAG ATCAT CCGCT TCGGC CAGAT GTTCT TCCCC

856 GAGTC GCGCG AGGAG ACCTA CTACC AGGAG TCGGC CGGCG TGGCC

901 GACCT GATCA CCACC TGCGC CGGCG GCCGC AACGT GAAGG TGGCC

946 CGCCT GATGG CCACC TCGGG CAAGG ACGCC TGGGA GTGCG AGAAG

991 GAGCT GCTGA ACGGC CAGTC GGCCC AGGGC CTGAT CACCT GCAAG

1036 GAGGT GCACG AGTGG CTGGA GACCT GCGGC TCGGT GGAGG ACTTC

1081 CCCCT GTTCG AGGCC GTGTA CCAGA TCGTG TACAA CAACT ACCCC

1126 ATGAA GAACC TGCCC GACAT GATCG AGGAG CTGGA CCTGC ACGAG

1171 GACTA A-CACGTG (*Pma*C I)-AAACGCG
