# Supplementary material for: Effect of overexpression of LPAAT and GPD1 on lipid synthesis and composition in green microalga Chlamydomonas reinhardtii
Source: J Appl Phycol. 2017 Dec 19;30(3):1711–9. doi: 10.1007/s10811-017-1349-2 (PMC5982436; doi:10.1007/s10811-017-1349-2)
Supplement: Supplementary file 2 — (DOCX 94.3 kb) [file 10811_2017_1349_MOESM2_ESM.docx]

**Supplementary Material 2:** **Growth of transgenic *C. reinhardtii*.**


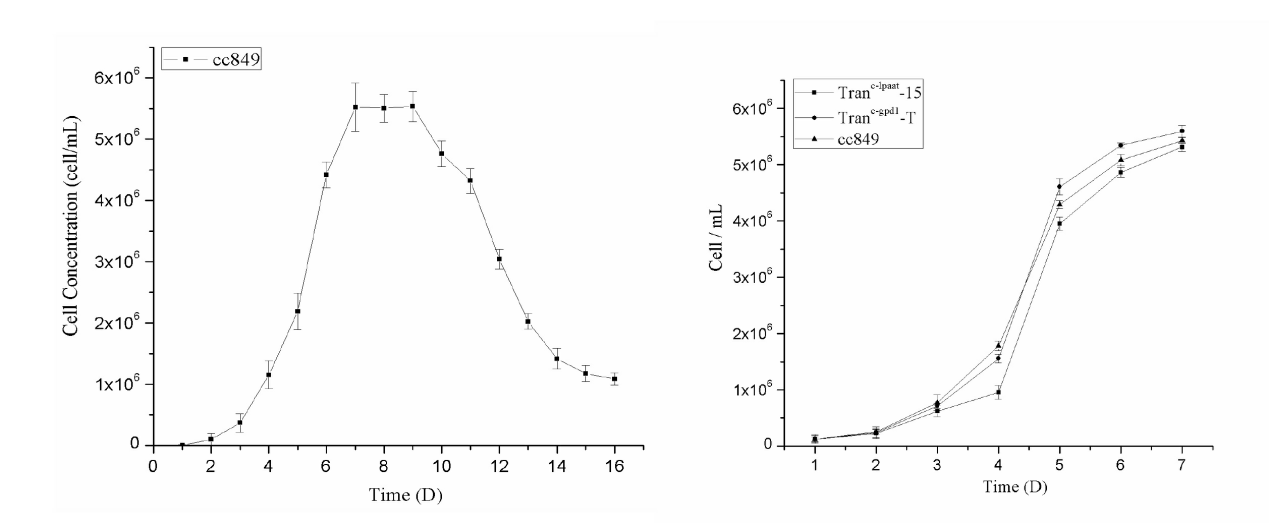


Tran^clpaat^ and Tran^cgpd1^ are algae introduce with *c-LPAAT* and *c-GPD1*, respectively. Alga CC-849 is wild type. Introduction of *c-LPAAT* and *c-GPD1* has no effect on the growth of transgenic algae.
