# Supplementary material for: Effect of overexpression of LPAAT and GPD1 on lipid synthesis and composition in green microalga Chlamydomonas reinhardtii
Source: J Appl Phycol. 2017 Dec 19;30(3):1711–9. doi: 10.1007/s10811-017-1349-2 (PMC5982436; doi:10.1007/s10811-017-1349-2)
Supplement: Supplementary file 3 — (DOCX 15.9 kb) [file 10811_2017_1349_MOESM3_ESM.docx]

**Supplementary Material：** Table S Change of algal fatty acid content and composition after one heat shock

|  |  |  |  |  |
| --- | --- | --- | --- | --- |
| \| Fatty acid \| Wild type \| pH124-1 \| pH124-2 \| pH124-3 \| \| --- \| --- \| --- \| --- \| --- \| \| (μg/mg DW) \| (μg/mg DW) \| (μg/mg DW) \| (μg/mg DW) \| \| 16:00 \| 15.05 \| 14.6 \| 15.1 \| 14.9 \| \| 16:01 \| 1.98 \| 1.88 \| 1.91 \| 1.99 \| \| 16:04 \| 27.71 \| 25.8 \| 28.51 \| 27.16 \| \| 18:00 \| 1.55 \| 1.51 \| 1.49 \| 1.55 \| \| 18:1t \| 1.1 \| 1.11 \| 1.08 \| 1.01 \| \| 18:2t \| 7.47 \| 7.51 \| 7.48 \| 7.44 \| \| 18:03 \| 9.83 \| 9.71 \| 9.88 \| 9.51 \| \| 18:3n3 \| 37.1 \| 36.5 \| 37.15 \| 39.1 \| \| Total \| 101.79 \| 98.62 \| 102.6 \| 102.66 \| \|  \|  \|  \|  \|  \| \| pH124-1,2,3 were transgenic algae transformed with pH124.Wild type was untransformed algae. Fatty acids in WT and pH124 transforments showed that there was no difference between them. \| \| \| \| \| | | | | |
